# Hyperacute Directional Hearing and Phonotactic Steering in the Cricket (*Gryllus bimaculatus* deGeer)

**DOI:** 10.1371/journal.pone.0015141

**Published:** 2010-12-08

**Authors:** Stefan Schöneich, Berthold Hedwig

**Affiliations:** Department of Zoology, University of Cambridge, Cambridge, United Kingdom; University of Arizona, United States of America

## Abstract

**Background:**

Auditory mate or prey localisation is central to the lifestyle of many animals and requires precise directional hearing. However, when the incident angle of sound approaches 0° azimuth, interaural time and intensity differences gradually vanish. This poses a demanding challenge to animals especially when interaural distances are small. To cope with these limitations imposed by the laws of acoustics, crickets employ a frequency tuned peripheral hearing system. Although this enhances auditory directionality the actual precision of directional hearing and phonotactic steering has never been studied in the behaviourally important frontal range.

**Principal Findings:**

Here we analysed the directionality of phonotaxis in female crickets (*Gryllus bimaculatus*) walking on an open-loop trackball system by measuring their steering accuracy towards male calling song presented at frontal angles of incidence. Within the range of ±30°, females reliably discriminated the side of acoustic stimulation, even when the sound source deviated by only 1° from the animal's length axis. Moreover, for angles of sound incidence between 1° and 6° the females precisely walked towards the sound source. Measuring the tympanic membrane oscillations of the front leg ears with a laser vibrometer revealed between 0° and 30° a linear increasing function of interaural amplitude differences with a slope of 0.4 dB/°. Auditory nerve recordings closely reflected these bilateral differences in afferent response latency and intensity that provide the physiological basis for precise auditory steering.

**Conclusions:**

Our experiments demonstrate that an insect hearing system based on a frequency-tuned pressure difference receiver achieves directional hyperacuity which easily rivals best directional hearing in mammals and birds. Moreover, this directional accuracy of the cricket's hearing system is reflected in the animal's phonotactic motor response.

## Introduction

For many animals directional hearing is fundamental to their lifestyle as it forms the basis for prey detection, predator avoidance or mate localisation [1]. Whereas vertebrate auditory systems generally rely on binaural sound level and arrival-time differences for the processing of directional information [2] these biophysical cues become tiny in animals like insects [3]. When the body is small compared to the wavelength of the perceived sound the directional performance of auditory systems is limited due to the lack of diffraction and minute interaural time differences [4].

Specific adaptations, however, are in place in some groups of insects [5]-[7]. Despite their small size, directional hearing is present to different degrees in flies, crickets, and bush-crickets [8], [9], [3]. Some of these insects possess microscale ears with astonishing functional properties. For example the hearing organ of the tachinid fly *Ormia ochracea* is sensitive to nanosecond interaural time differences and allows discrimination of sound angles as small as 1-2° [10], [11].

In bush-crickets and crickets a different type of hearing organ provides the basis for directional hearing. They employ a pressure gradient system that has an inherent directional sensitivity [12], [13]. Hearing organs are located in the front legs [14] and sound acts on each auditory organ via tympanic membranes in the tibia and via a specialized auditory trachea with large openings in the anterior body wall [15]. Crickets in particular are a neuroethological model system for auditory processing and orientation in insects as females use acoustic cues of the males' calling song to find mates [16], [17]. When walking on a closed-loop trackball system that electro-mechanically compensated the animals' walking movements, the phonotactic paths of field crickets (*G. bimaculatus, G. campestris*) meandered by 30°-60° around the frontal midline [18], [19]. Directional orientation in a Y-maze indicated that these crickets are not able to discriminate the side of sound incidence when the source deviates less than 25° from their longitudinal axis [20]. Supported by early biophysical analysis of the hearing system these findings led to the conclusion that *G. bimaculatus* face a frontal area of uncertainty covering ±25° azimuth in which they have insufficient directional sensitivity to steer directly towards the sound source [20], [21]. Behavioural studies in *Teleogryllus oceanicus*, however, reported directional acuity of 10-14° for side discrimination in female's phonotactic responses [22]-[24].

When sound stimuli were presented to crickets either from 30° ipsilateral or contralateral a bilateral difference in response amplitude corresponding to about 10 dB sound intensity was measured by recording the summed activity of the auditory afferents [25] and also by recent laservibrometric measurements of tympanic membrane oscillations in *Gryllus bimaculatus* [26]-[28]. Moreover, *G. bimaculatus* exhibit a high sensitivity towards interaural differences in sound intensity and orient towards the louder of two sound sources at bilateral intensity differences of 1 dB and less [29, and unpublished data]. Given this high degree of intensity discrimination a frontal bilateral auditory intensity gradient of 10 dB over 30° appears to be in contradiction to the supposed area of directional uncertainty in these animals.

We tested *G. bimaculatus* females walking on an open-loop trackball system and demonstrate that they localise the azimuth angle of a speaker presenting a male calling song between 1° and 30° off the animal's longitudinal axis. We analysed the peripheral coding of the azimuth of sound and show that a frequency-tuned pressure gradient receiver as it operates in crickets [25], [28] is efficient for hyperacute processing of directional auditory information.

## Materials and Methods

### Animals

**Figure 1:**
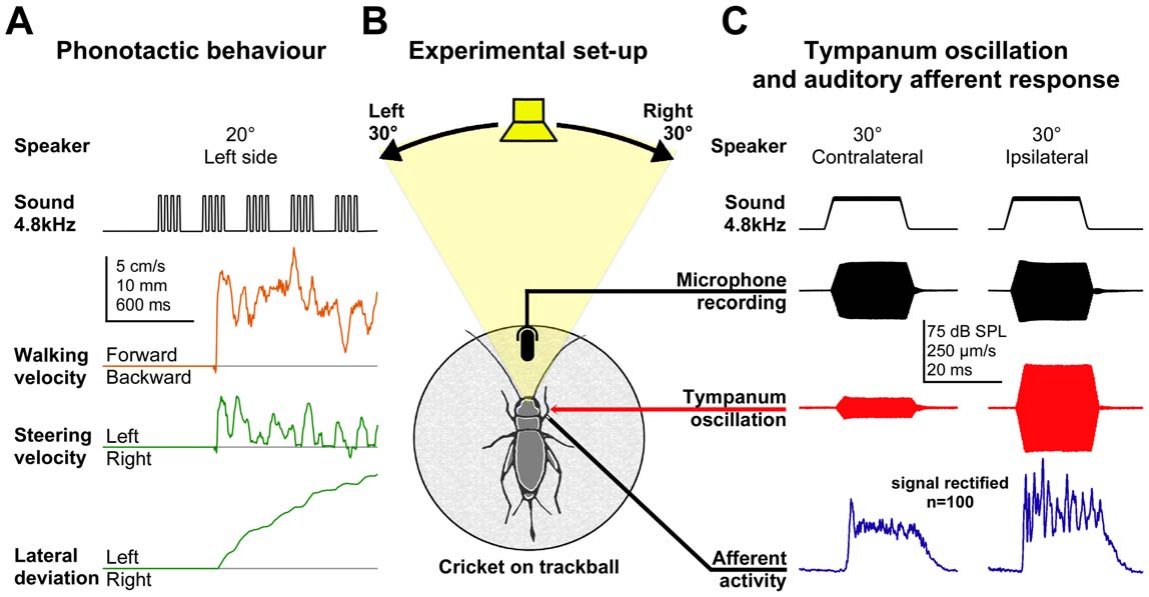
Experimental design. (A, B): Tethered crickets were walking on a trackball while male calling song was presented from different angles of incidence between 0° and ±30°. Speaker position and acoustic stimulation was computer controlled. The rotations of the trackball were monitored and provided the walking velocity and the steering velocity of the animal. The steering velocity was integrated to calculate the lateral deviation in response to acoustic stimulation. (B, C): Laservibrometric and neurophysiological recordings. Crickets were tethered into a wireframe and placed on a trackball in walking posture. Traces show sound pulses presented from 30° ipsilateral and contralateral, sound level recordings above the cricket, tympanic membrane oscillations and the auditory afferent responses. The afferent activity was full wave rectified and 100 responses were averaged.

Female crickets (*Gryllus bimaculatus* deGeer) were isolated as last instars from the cricket colony at the Department of Zoology, Cambridge. Animals were reared individually and fed on a protein and fat rich diet and water. Only females with intact silvery-white tympana were selected for experiments. One week after their final moult a tether was fixed to the third thoracic tergite, close to the animal's centre of gravity. For testing the precision of phonotactic steering females were positioned on top of an open-loop trackball system (Fig. 1A,B). During walking the tethered cricket rotated the trackball. The rotational movements of the trackball were measured with an optical mouse sensor (ADNS-2051, 2D Optical Mouse Sensor; Agilent, Farnell Electronics, Oberhaching, Germany) and provided the forward walking and lateral steering velocities of the animal. Velocity data were integrated to calculate the animal's forward walking distance and the lateral deviation for any sound sequence tested [29], [30]. Sound intensity was calibrated with a ¼" free field microphone at the position of the cricket and adjusted to 75 dB SPL (Brüel and Kjær Nærum, Denmark, amplifier type 2610, microphone type 4191).

### Acoustic stimulation

Experiments were performed in a dark and sound proof chamber lined with sound damping tiles (Sonex 65/125; Illbruck, Bodenwöhr, Germany). Background noise level at the position of the crickets was 38 dB SPL, rel. 10^–5^ N m^2^ (band-pass filter 200 Hz - 200 kHz). Models of male calling song were computer generated using audio software (Cool Edit 2000, Syntrillium, Phoenix, USA, now Adobe Audition). Songs had a carrier frequency of 4.8 kHz, 4 pulses (20 ms duration, incl. 2 ms rise and fall time, 22 ms intervals) repeated at 300 ms and were presented at 75 dB SPL.

The speaker (Sinus live, Neo13s, Conrad Electronics, Hirschau, Germany) was level with the cricket. It was attached to a 57.3 cm long lever fitted to the axis of a stepper motor (type 4490H048B K1155, controller MCNL3006S; Faulhaber GmbH, Schönaich, Germany) so that a rotation by 1° corresponded to a displacement of 1 cm. The motor axis was aligned with the centre of the trackball. The control software allowed positioning the speaker with an accuracy of less than 0.5° at any angle to the cricket's length axis. Speaker movements were monitored with a 360° smart position sensor (resolution of 0.5°, model 601-1045 Vishay S.A., Nice France) coupled to the motor axis. We define a speaker position of 0° as frontal to the animal.

### Testing directional sensitivity

The crickets were placed on the trackball with their body length axis adjusted in line with the 0° speaker position. Their phonotactic responsiveness was checked by presenting calling song from 30° to the left and right. If females responded with phonotaxis during the consecutive tests the speaker then was positioned alternating to the left and right side of the animal to horizontal angles of 0°-5° in 1° intervals, to 6°-12° in 2° intervals, to 15°, 20°, 30° and finally to 0° again. At each angle calling song was presented for 30 s, then a silent interval of 10 s followed in which the speaker moved to the next position. The complete test sequence lasted 18 min. Fifteen phonotactic active females were selected and each of them tested 3 times on consecutive days. We analysed the relationship between phonotactic response and incident angle for each female. To eliminate any bias in walking direction the respective left and right steering responses were pooled. The forward distance and the lateral deviation for each speaker position were calculated relative to the animals mean forward and steering response over all angles tested, which was set to 100%. Normalizing to the mean as compared to the maximum response takes the overall performance of the animal into consideration. The walking vector angle, giving the direction of the animal's walk, was calculated between the start point and the virtual end point of the cricket's path after 30 s, relative to the 0° speaker position. In a second group of animals (n = 15) stimulation angles (0°, 2°, 4°, 6°, 8°, 10° and 20°) were randomly varied in order and direction to control for any effects due to the order of stimulus presentation.

### Laservibrometric measurements and auditory nerve recordings

Female crickets were cold-anaesthetised (20 min at 4°C) and the thoracic ganglia were carefully removed to reduce spontaneous motor activity. A metal rod (2 mm Ø) was waxed dorsally onto the pronotum and wings. With small droplets of melted wax all legs were tethered to 0.3 mm copper wires extending from the rod and then the cricket was placed on a trackball in walking position (Fig. 1B,C). Glass nanobeads (3 µm Ø; 0.36 µg weight) extracted from a HPLC column were placed on the centre of the posterior tympanic membrane of the left front leg to increase its reflectance. A laservibrometer (Polytec PDV 100, Waldbronn, Germany) with a maximum sensitivity range of 1.25 (mm/s)/V was positioned 33 cm behind the animal. The He-Ne laser was adjusted and focussed on the centre of the tympanum using a XY-stage (Model 100cr, Siskuyo Design Instruments, Grants Pass, OR, USA). Tympanic membrane oscillations during acoustic stimulation were measured at the same speaker positions as during the behavioural experiments and additionally at 25°. The laservibrometer signal was band-pass filtered (100 Hz HP and 5 kHz LP i.e. upper and lower frequency for precise amplitude measurements) and its RMS (root mean square) calculated online by an integrated circuit (Analog Devices Type 637, Farnell Electronics, UK). For analysis the stimulus related RMS area over background activity was calculated in mV/s. At all speaker positions 100 sound pulses (4.8 kHz, 20 ms duration including 2 ms rise and fall time, 80 ms interval) were presented. As a reference the response of the tympanic membrane oscillations and the auditory nerve activity were measured with a speaker position at 0° azimuth for sound intensities between 64 and 80 dB SPL in steps of 2 dB. Sound intensity was checked with a ¼" free field microphone positioned 2 cm above the animal and adjusted to 75 dB SPL.

In 10 experiments we measured the oscillations of the left tympanic membrane and in 4 of these experiments we recorded at the same time the summed afferent activity of the associated ear. The tympanal nerve [14] was exposed at the distal femur and its activity recorded using a platinum wire hook electrode (50 µm Ø) and standard extracellular recording techniques. Signals were amplified using a differential AC amplifier and 300 Hz - 5 kHz band-pass filtered (Model AC 1700, A-M Systems, Carlsborg, WA, USA). For quantitative analysis the afferent signal was full-wave rectified before averaging the auditory response to all 100 sound pulses at each speaker position (Fig. 1C). From these averages as a measure of the overall afferent response the signal area over background activity was calculated in mV/s [31]. The response latency was measured for each sound pulse in the original recording trace and thereafter the average latency was calculated. Ipsilateral and contralateral are used with reference to the recorded ear.

### Data sampling

All signals were sampled at 30 kHz per channel with an AD board (National Instruments PCI-Mio 16-E-4; National Instruments Newbury, UK) controlled under LabView 5.01 and stored on the hard disk of a PC. Data analysis was performed off-line with custom written software [32]. For further statistical analysis and calculation of histograms data was imported to a spreadsheet program (Microsoft Excel). Mean values are given ±SD.

## Results

### Directional steering during phonotactic behaviour

**Figure 2:**
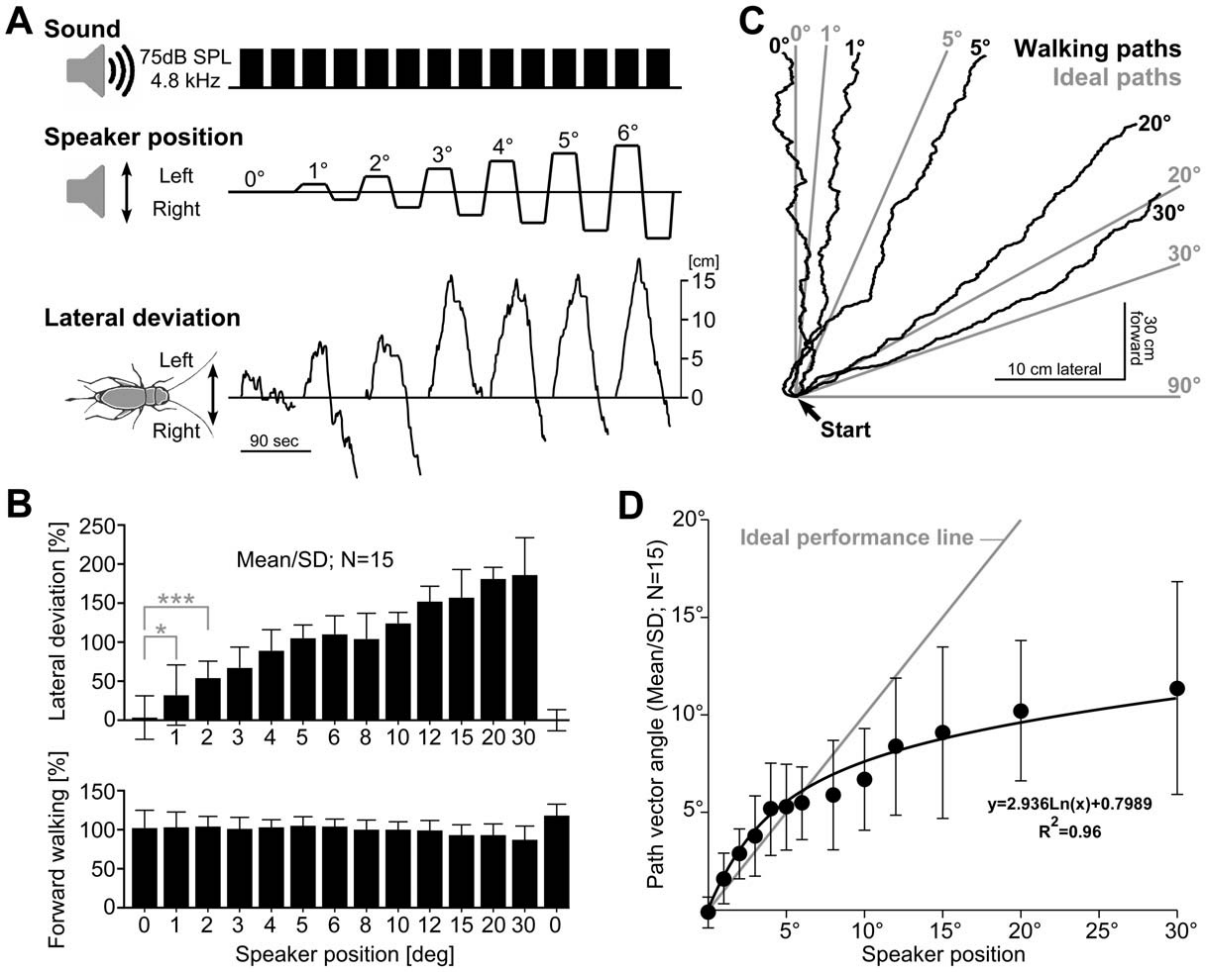
Phonotactic behaviour. (A): Directional acoustic stimulation between 0° and ±6° azimuth and cricket steering responses. At each speaker position calling song was presented for 30 s at 75 dB SPL. Steering response (lateral deviation) increased with increasing speaker azimuth. (B): Lateral deviation and forward walking response for different speaker positions. Lateral deviation increased with increasing stimulation angles. (A, B): Clear steering towards the stimulation side occurred already at ±1° azimuth; *t*-Test: *p<0.05; ***p<0.005. (C): Combining lateral deviation (x-axis) and forward movement (y-axis) revealed individual walking paths of a cricket over 30 s. At small stimulus angles the cricket slightly oversteered and for larger angles they steered less in comparison to ideal path lines. Note the different scaling for forward and lateral walking component. (D): Relation between stimulation angle and the walking vector angle. Relative to the ideal performance line the animals slightly oversteered for speaker positions smaller than 6° and they increasingly understeered for positions larger than 6°. The trendline corresponds to a logarithmic function. (B, D): Data pooled from 15 animals.

On the trackball the female crickets walked spontaneously or started walking upon hearing the calling song. When the song was presented exactly from the front (0° speaker position) the females consistently walked forward, and showed only minor stochastically lateral deviations to the left or right (Fig. 2A,C). Already at 1° stimulus angle, however, 67% of the crickets consistently showed a directed deviation towards the side of acoustic stimulation. At angles equal or larger than 2° all females exhibited clear steering towards the speaker side and their steering response increased with increasing angle of incidence up to 30°, the maximum angle tested. The steering response towards the acoustic stimulus presented at the same angle from the left and right side was not always symmetrical (Fig. 2A). Although this female showed a symmetrical response at 3° it overall walked somewhat more to the right. Such a lateral bias occurred in several animals; it could change in amount and direction from day to day or even during the same session. Presenting the calling song for each given angle consecutively from the left and right side and taking the mean value of the corresponding steering responses cancelled the effects of any lateral bias. We pooled the relative steering responses and relative forward walking distance of all 15 individuals for each stimulus angle; 100% relative steering response corresponding to a mean lateral deviation of 13.6 cm/min and 100% forward walking corresponding to 156.0 cm/min (Fig. 2B). At frontal speaker position the mean lateral deviation and path vector angle were minimum (0.2±2.4 cm/min; 0.3±1.6°). The steering response of all tested animals was significantly directed towards the side of acoustic stimulation for all stimulus angles. Even the steering response to 1° azimuth (lateral deviation: 4.0±3.9 cm/min; path vector angle: 1.6±1.3°) was significantly different to the response to 0° (*t*-Test: p = 0.01). As the angle of incidence increased to 5° the lateral steering response increased almost linearly by about 18% per degree (R^2^ = 0.99 for linear fit). Up to 4° the steering response between adjacent angles was significantly different (*t*-Test p<0.05). For larger angles the steering responses gradually increased, although they were not all significantly different. At 30°, the largest angle tested, the relative steering response was 186%, corresponding to 25.3 cm/min.

The overall distance that the animals walked forward, as determined by integrating their forward velocity, gradually decreased with increasing angle of sound incidence down to 87% (139.5 cm/min) at 30° (Fig. 2B). This decrease was not due to fatigue as testing the response to 0° at the end of the paradigm revealed a slightly larger forward distance as at the beginning of the experiment (mean before test: 158 cm/min; mean after test: 182 cm/min). The decrease in forward walking was moreover a consequence of the stronger lateral steering as the crickets increasingly turned towards the speaker [29].

By integrating the forward velocity and the lateral velocity of the animals we calculated the 2-dimensional individual walking paths of a steering cricket (Fig. 2C). For each 30 s trial of each animal we further determined the path vector angle, i.e. the angular deviation from a straight forward path and plotted the pooled data against the speaker position (Fig. 2D). In a perfect system the steering angle would match the stimulus angle. For small stimulation angles (1°-5°) the steering angle closely followed the ideal performance line but the animals tended to oversteer slightly. In the range of 8°-30° they increasingly understeered relative to the angle of sound incidence as the mean steering at 30° was only 11.3°±5.6°. This characteristic tendency of the steering behaviour is also reflected in the 2-dimensional plots of individual walks (Fig. 2C) as the real paths correspondingly deviated from the ideal paths.

Furthermore we calculated the accuracy of course maintenance by dividing the vector length (i.e. the distance between the start- and endpoint of a walk) by the actual path length of the animal, with a value of 1 indicating walking perfectly along a straight line. The walking accuracy averaged over all animals and all tests was 0.92±0.03. Between animals the mean accuracy of course maintenance varied between 0.88±0.03 and 0.95±0.01, which is a statistically significant difference (*t*-Test, p<0.0001). For stimulation from 0° and 30° the pooled data of all 15 animals were not significantly different with values of 0.91±0.04 and 0.92±0.03, respectively. Thus, the accuracy of course maintenance varied considerably between different animals, but did not significantly depend on the incident angle of the acoustic stimulation.

Since our sequential stimulus paradigm tested the smallest angles first, the animals' behaviour indicated clear responses to the incidence of sound rather than an adaption to the bilateral pattern of the stimulation sequence. We also tested 15 animals with a randomly arranged sequence of stimulus angles. These tests gave the same steering responses and we therefore exclude that learning the testing schedule had an influence on the animals' directional steering responses.

### Directional responses of the posterior tympanic membrane

In a different set of experiments we analysed the biophysical cues underlying phonotactic steering. Crickets were tethered to a body-sized wire frame and positioned on the trackball in walking posture (Fig. 1B,C). The oscillations of the left posterior tympanum were measured with a laservibrometer. Sound stimuli were presented from angles between 0° and 30° from the ipsilateral and contralateral side. The recordings in Fig. 1C show the sound signal, the tympanic membrane oscillations and the corresponding auditory afferent activity. For stimuli presented from 30° ipsilateral and contralateral, respectively, they demonstrate a clear difference in the response of the tympanic membrane and the auditory afferents.

**Figure 3:**
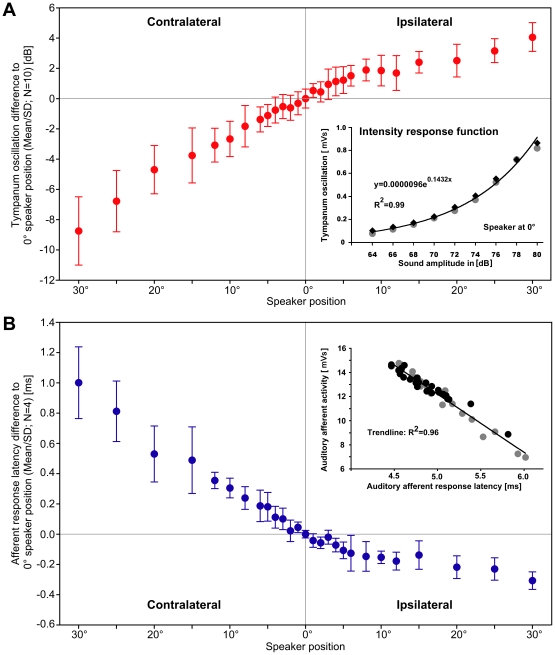
Characteristic plots of directionality. (A): Amplitude changes of the tympanic membrane oscillations relative to the response at 0° speaker position. In the range of ±30° the response amplitude gradually increased by 4.1 dB for ipsilateral and it decreased by 8.8 dB for contralateral stimulation. (Inset A): The intensity response function of tympanic membrane oscillations for frontal sound stimuli presented at 64-80 dB SPL (gray, black: before and after directional test). (B): Changes in the latency of the summed afferent response relative to the latency at 0° speaker position. In the range of ±30° the response latency gradually decreased by 0.3 ms for ipsilateral and it linearly increased by 1.0 ms for contralateral stimulation. (Inset B): Correlation between auditory afferent response latency and afferent activity with a linear regression line fitting the data points. Each data point is mean response of 100 stimulus repetitions. Black dots represent data from directional tests. Gray dots represent data from frontal acoustic stimulation while the sound intensity was varied in 2 dB steps between 64 to 80 dB SPL. Each sound intensity was tested before and also after the directional test.

Before and after the directional tests, sound pulses of 64-80 dB SPL (in 2 dB steps) were presented from the frontal 0° direction. Plotting the resulting intensity response function for the tympanic membrane oscillations demonstrated that the amplitude of its oscillations increased exponentially with increasing sound intensity. The measurements indicated the quality of the laser signal over time as the intensity response function obtained before and after the directional tests yielded the same values (inset Fig. 3A). Consecutively these data were used to scale the amplitude of tympanic membrane oscillations at different angles of incidence with the intensity response function to frontal acoustic stimulation. The relative change in the amplitude of tympanic membrane oscillations exhibited a characteristic asymmetric course across the angles of incidence tested (Fig. 3A). When the speaker moved from the frontal position to 30° ipsilateral the response increased by 4.1±1.0 dB in a non-linear way with a steeper increase up to 6° and a more gradual increase from 8° to 30°. When the speaker moved from 0° frontal to 30° contralateral the response amplitude linearly decreased over the whole range by 8.8±2.3 dB.

### Tympanic nerve activity during directional stimulation

For successful auditory steering to be achieved, the mechanical responses of the hearing organs need to be precisely encoded. In crickets from each ear a population of 45-60 primary afferents forward auditory activity towards the central nervous system for further processing. Using extracellular recordings of the afferent axons in the tympanal nerve [14] we analysed any changes in auditory evoked neural activity related to the angle of acoustic stimulation (Fig. 3B). In 4 animals we obtained stable long-term recordings of the afferent activity simultaneously with measurements of the tympanic membrane oscillations during directional acoustic stimulation. In these long-term recordings the response latency provided a most robust parameter and was therefore used for the quantitative analysis. For the directional tests the sound intensity was kept at 75 dB SPL and response latency changes for each speaker position were calculated relative to frontal stimulation (Fig. 3B). The decrease in latency was moderate for increasing ipsilateral stimulation angles but pronounced at the contralateral side. Latency decreased by 0.31±0.06 ms when the speaker moved from frontal to 30° ipsilateral and it increased by 1.00±0.24 ms, when the speaker was moved from the front to 30° at the contralateral side. Similar to the tympanic membrane oscillations the relative change in the afferent latency exhibited a characteristic asymmetric course across the stimulation angles. We also analysed the afferent response latencies to sound pulses in the range of 64 to 80 dB SPL (in 2 dB steps) with a frontal speaker position in order to calibrate the latency measurements, providing a latency difference of 1.4 ms for stimulation with 64 and 80 dB SPL. All recordings demonstrated a close and linear correlation between afferent response latency and activity (inset Fig. 3B). Therefore the angle of sound incidence will be reflected in the afferent activity as well, however with an inverted corresponding curve [33].

### Interaural response differences underlying directional hearing and steering

**Figure 4:**
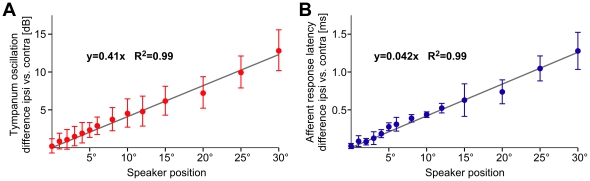
Interaural response difference functions. (A): Differences in the amplitude of tympanic membrane oscillations as calculated for corresponding bilateral speaker positions. A linear regression line fit the data points. (B): Differences in the response latency of the summed afferent recording as calculated for corresponding bilateral speaker positions. A linear regression line fits the data points.

Based on the characteristic biophysical and neurophysiological data sets we determined the directional response functions of the cricket auditory system in the frontal range from 0° to 30°. Assuming a symmetric response function of both ears we calculated the bilateral response differences at each angle tested (Fig. 4A,B).

For tympanic membrane oscillations the binaural response difference was 12.9±2.7 dB at 30°. All values between 0° and 30° were closely fitted by the linear regression function y = 0.41× (R^2^ = 0.99). Therefore tympanic membrane oscillations in the frontal range of ±30° correspond to interaural intensity difference of 0.41 dB/°. For afferent latencies the interaural difference at 30° reached 1.3±0.3 ms resulting in a gradient for bilateral latency differences of 42 µs/°. Again values for all angles tested were closely fitted by a linear regression function (y = 0.042×; R^2^ = 0.99). From our calibration data we can estimate that a latency difference of 1.3±0.3 ms corresponded to a difference in sound amplitude of 10-16 dB SPL in different animals. Thus in the frontal range of ±30° the cricket auditory system provides the female with a linear gradient of about 0.4 dB/° to localise and approach a singing male.

## Discussion

The results presented here shed new light on the precision of phonotactic orientation in the field cricket *Gryllus bimaculatus*. They go well beyond previous conclusions and reveal hyperacute directional hearing in these animals. Video recordings by Rheinlaender and Blätgen [20] of freely walking *G. bimaculatus* demonstrated that the females approached a male calling song on a zigzag-course meandering around the straight target direction and made most of their incorrect turns for target angles below 20°. Oscillations by 30°-60° amplitude around the direction of the sound source were consistently recorded in female crickets walking on a closed-loop trackball system that electro-mechanically compensated the animals' walking movements [34], [35]. However, with such a compensated trackball system walking cannot be entirely natural as when the leg forces accelerate the cricket forward, the sphere counter-accelerates the animal with a delay causing abnormal sensory feedback [18]. When tested in a Y-maze crickets were not able to reliably make correct decisions if the angle of sound incidence was smaller than 25°. Together with early laservibrometric measurements of directional responses of the tympanic membrane [36] these observations resulted in the conclusion that crickets face a ±25° frontal area of directional ambiguity, in which they have no reliable information on the incidence of sound [20], [21], [26].

It has been proposed that freely walking crickets overcome this ambiguity by meandering around the direction of sound incidence and consecutively steering to the side of the ear stronger activated [37], [38]. However, for tethered females walking under open-loop conditions, in which the animal's steering reactions have no effect on the angle of incidence, precise steering responses towards small stimulus angles could not have been expected given a ±25° frontal range of directional uncertainty. Thus the steering data presented here may require considering alternative explanations for the meandering walking paths observed in animals walking freely or under compensated closed-loop conditions.

In our experiments the females showed highly accurate directional steering. Already at 1° they correctly discriminated the side of acoustic stimulation. Furthermore at small angles of 1-6° the animals not just recognised the side of acoustic stimulation; but they clearly adjusted their phonotactic response to the angle of incidence. From 6° to 30° the steering angle further increased, but the animals increasingly understeered relative to the angle of sound incidence (Fig. 2D) despite the linear sensory resolution of the sound direction (Fig. 4). This indicates that for angles larger than 10° crickets walking under closed loop conditions would need several steps to align towards the sound source. Once aligned precise corrective steering reactions to small deviations from the direct course allows a highly efficient phonotactic approach. Since our data indicate that auditory orientation in crickets does not necessitate meandering walking paths as observed in other studies [34], [35], [18] the different experimental conditions may need scrutinizing. In contrast to freely walking crickets, tethering the animals on top of a trackball system may have had two major consequences. As the animals walked under open-loop conditions steering manoeuvres did not alter the orientation of the animals within the sound field so that sound stimuli were always perceived under identical acoustic conditions. Further even minute auditory steering responses were added up over the testing period revealing the directional performance of the cricket's phonotactic steering system under favourable conditions for auditory perception and sensory-to-motor processing. While walking on the ground through vegetation crickets have to deal with considerable sound scatter leading to transient and local signal distortion [39]. Under these complex acoustical conditions the animals will clearly benefit from an auditory system with high directional precision.

The accuracy of phonotactic steering towards small angles of incidence requires that the left and right ear respond sufficiently different to the directional sound signals. Furthermore these differences have to be reflected in the auditory afferent activity which is forwarded towards the central auditory pathway. Our laservibrometric measurements of the tympanic membrane oscillations and our tympanal nerve recordings demonstrated an interaural intensity difference corresponding to 12.8 dB at 30° sound incidence. This value and the overall characteristic change in the frontal range (Fig. 3) is similar to recent laservibrometric measurements by Michelsen & Löhe [27] and previous auditory afferent recordings by Boyd & Lewis [25]. At 30° sound incidence a 2-3 dB response increase was reported for the ipsilateral ear whereas at the contralateral ear the response decreased by 7-9 dB, both values relative to frontal acoustic stimulation. Different to previous experiments we measured the characteristic response curves in the frontal range of the cricket with a fine grid of angular resolution (Fig. 3). To increase the reflectance of the tympanic membrane we used glass nanobeads with dimensions (3 µm Ø; 0.36 µg weight) in the range of naturally occurring fine dust particles. This procedure added a minute mass to the tympanum which could have slightly damped its acoustically evoked oscillation. If at all our measurements may have underestimated the absolute response amplitudes, but as we analysed relative amplitude changes we consider such effects as negligible. Our data demonstrate that at 4.8 kHz, which is the carrier frequency of the male calling song [40], *G. bimaculatus* females can use a linear slope of 0.41 dB/° interaural signal difference in the frontal area for a precisely directed phonotactic approach.

The gradient of interaural intensity differences is generated by the acoustic properties of the cricket's frequency-tuned peripheral hearing system [15], [25], [27] as the sound intensity at both sides of the cricket's body actually differs only by 1.3 dB when stimulated from 30° [26]. The interaural intensity differences were closely reflected in the response latencies of the auditory afferent activity. The overall bilateral latency difference at ±30° was 1.28 ms whereas the actual interaural difference in sound arrival time is less than 15 µs. The linear latency gradient of 42 µs/° in the frontal range thus reflects almost exclusively the gradient of interaural intensity differences [33], [41]. Interaural amplitude and latency differences of the afferent activity are forwarded to the central nervous system for further bilateral contrast enhancement by reciprocal inhibition [42]-[45]. Many auditory systems use smaller afferent latency differences than the cricket for directional orientation [46]. An acoustically orienting fly even exploits a gradient of only 3.5 µs/° over a range of ±30° azimuth [11].

Our results reveal the acoustic orientation of the cricket *G. bimaculatus* as one of the most precise among invertebrates and place it at the same level to the achievements of vertebrate directional hearing. Bush-crickets can reliably turn to a sound signal when the angle of incidence is at least in the range of 5°-10° [47], [48]. At a minimum azimuth angle of 10° degrees grasshoppers correctly turn towards the side of acoustic stimulation [49]. Among insects only the fly *Ormia ochracea* achieves a similar hyperacute directional hearing as they reliably orientate to sound sources deviating by 1-2° in azimuth [11]. In a species of ultrasonic communicating frogs males localize calling females with an acuity of just 1° [50] and the well-studied barn owl has a minimum audible angle in its frontal region of 1.5°-2° [51]. Mammals vary considerably in their ability to localise the precise direction of a frontal sound source [52]. Only few species have been reported to achieve an accuracy comparable to the cricket: humans can recognise azimuth deviations of 1-2° [53], echolocating bats discriminate angles of sound incidence with 1.5° difference [54], elephants 1.0° [55] and dolphins even 0.7°-0.8° [56].

The highly accurate phonotactic steering of crickets demonstrates that at the carrier frequency of the male calling song even minute interaural differences in the sound signal provide significant directional information. In a frequency tuned pressure difference receiver, such as the cricket auditory pathway, phase differences generated within the auditory trachea have been shown to be of crucial importance [27], [28]. This invites further experimental analysis at the level of the auditory system and phonotactic behaviour to illuminate the function of internal phase differences for precise directional orientation. The hyperacuity of phonotactic behaviour in the cricket points towards specific adaptations at the biophysical level of the hearing organ. However, so far the mechanisms transforming sound waves into tympanum oscillations and then into auditory afferent activity have not been revealed in detail.

## References

[1] Bradbury JW, Vehrencamp SL (1998). Principles of animal communication..

[2] Warren RM (1999). Auditory perception: A new analysis and synthesis..

[3] Hennig RM, Franz A, Stumpner A (2004). Processing of auditory information in insects.. Microsc Res Tech.

[4] Michelsen A, Webster DM, Fay RR, Popper AN (1992). Hearing and sound communication in small animals: evolutionary adaptations to the laws of physics.. The evolutionary biology of hearing.

[5] Hoy RR, Robert D (1996). Tympanal hearing in insects.. Ann Rev Entomol.

[6] Yager DD (1999). Structure development and evolution of insect auditory systems.. Microsc Res Tech.

[7] Stumpner A, von Helversen D (2001). Evolution and function of auditory systems in insects.. Naturwissenschaften.

[8] Robert D, Amoroso J, Hoy RR (1992). The evolutionary convergence of hearing in a parasoid fly and its cricket host.. Science.

[9] Hoy R, Nolen T, Brodfuehrer P (1989). The neuroethology of acoustic startle and escape in flying insects.. J Exp Biol.

[10] Robert D (2001). Innovative biomechanics for directional hearing in small flies.. Biol Bull.

[11] Mason AC, Oshinsky ML, Hoy RR (2001). Hyperacute directional hearing in a microscale auditory system.. Nature.

[12] Autrum H (1940). Über Lautäusserungen und Schallwahrnehmung bei Arthropoden. II. Das Richtungshören von *Locusta* und der Versuch einer Hörtheorie für Tympanalorgane vom Locustidentyp.. J Comp Physiol A.

[13] Michelsen A, Larsen ON (2008). Pressure difference receiving ears.. Bioinspir Biomim.

[14] Nocke H (1972). Physiological aspects of sound communication in crickets (*Gryllus campestris* L.).. J Comp Physiol A.

[15] Larsen ON, Michelsen A (1978). Biophysics of the Ensiferean ear. III. The cricket ear as a four input system.. J Comp Physiol A.

[16] Hoy RR (1978). Acoustic communication in crickets a model system of feature detection.. Fed Proc.

[17] Huber F, Moore TE, Loher W (1989). Cricket behaviour and neurobiology..

[18] Weber T, Thorson J, Huber F (1981). Auditory behavior of the cricket: I. Dynamics of compensated walking and discrimination paradigms on the Kramer treadmill.. J Comp Physiol A.

[19] Weber T, Thorson J, Huber F, Moore TE, Loher W (1989). Phonotactic behavior of walking crickets.. Cricket behaviour and neurobiology.

[20] Rheinlaender J, Blätgen G (1982). The precision of auditory lateralization in the cricket *Gryllus bimaculatus*.. Physiol Entomol.

[21] Larsen ON, Kleindienst HU, Michelsen A, Huber F, Moore TE, Loher W (1989). Biophysical aspects of sound reception.. Cricket behaviour and neurobiology.

[22] Bailey WJ, Thomson P (1977). Acoustic orientation in the cricket *Teleogryllus oceanicus* (Le Guillou).. J Exp Biol.

[23] Oldfield BP (1980). Accuracy of orientation in female crickets, *Teleogryllus oceanicus* (Gryllidae): Dependence on song spectrum.. J Comp Physiol A.

[24] Pollack GS, Plourde N (1982). Directionality of acoustic orientation in flying crickets.. J Comp Physiol A.

[25] Boyd P, Lewis B (1983). Peripheral auditory directionality in the cricket (*Gryllus campestris* L., *Teleogryllus oceanicus* Le Guillou).. J Comp Physiol A.

[26] Michelsen A, Popov AV, Lewis B (1994). Physics of directional hearing in the cricket *Gryllus bimaculatus*.. J Comp Physiol A.

[27] Michelsen A, Löhe G (1995). Tuned directionality in cricket ears.. Nature.

[28] Michelsen A (1998). The tuned cricket.. News Physiol Sci.

[29] Hedwig B, Poulet JFA (2005). Mechanisms underlying phonotactic steering in the cricket *Gryllus bimaculatus* revealed with a fast trackball system.. J Exp Biol.

[30] Hedwig B, Poulet JFA (2004). Complex auditory behaviour emerges from simple reactive steering.. Nature.

[31] Hedwig B (1989). Modulation of auditory information processing in tethered flying locusts.. J Comp Physiol A.

[32] Knepper M, Hedwig B (1997). NEUROLAB, a PC-program for the processing of neurobiological data.. Comput Methods Programs Biomed.

[33] Mörchen A, Rheinlaender J, Schwartzkopff J (1978). Latency shift in insect auditory nerve fibers: A neuronal time cue of sound direction.. Naturwissenschaften.

[34] Wendler G, Dambach M, Schmitz B, Scharstein H (1980). Analysis of the acoustic orientation behavior in crickets, *Gryllus campestris* L.. Naturwissenschaften.

[35] Schmitz B, Scharstein H, Wendler G (1982). Phonotaxis in *Gryllus campestris* L (Orthoptera, *Gryllidae*): I. Mechanism of acoustic orientation in intact female crickets.. J Comp Physiol.

[36] Larsen ON, Surlykke A, Michelsen A (1984). Directionality of the cricket ear: A property of the tympanal membrane.. Naturwissenschaften.

[37] Thorson J, Weber T, Huber F (1982). Auditory behavior of the cricket: II. Simplicity of calling-song recognition in *Gryllus*, and anomalous phonotaxis at abnormal carrier frequencies.. J Comp Physiol A.

[38] Schildberger K, Schildberger K, Elsner N (1994). The auditory pathway of crickets: Adaptations for intraspecific acoustic communication.. Neural basis of behavioural adaptations.

[39] Kostarakos K, Römer H (2010). Sound transmission and directional hearing in field crickets: Neurophysiological studies outdoors.. J Comp Physiol A.

[40] Kostarakos K, Hennig MR, Römer H (2009). Two matched filters and the evolution of mating signals in four species of cricket.. Frontiers in Zoology.

[41] Mason AC, Faure PA (2004). The physiology of insect auditory afferents.. Microscop Res Tech.

[42] Wiese K, Eilts K (1985). Evidence for matched frequency dependence of bilateral inhibition in the auditory pathway of *Gryllus bimaculatus*.. Zool Jb Physiol.

[43] Horseman G, Huber F (1994). Sound localisation in crickets. I. Contralateral inhibition of an ascending interneurone (AN1) in the cricket *Gryllus bimaculatus*.. J Comp Physiol A.

[44] Faulkes Z, Pollack GS (2000). Effects of inhibitory timing on contrast enhancement in auditory circuits in crickets (*Teleogryllus oceanicus*).. J Neurophysiol.

[45] Pollack GS (2003). Sensory cues for sound localization in the cricket *Teleogryllus oceanicus*: interaural difference in response strength versus interaural latency difference.. J Comp Physiol A.

[46] Carr CE, MacLeod KM (2010). Microseconds matter.. PLoS Biol.

[47] Bailey WJ, Stephen RO (1984). Auditory acuity in the orientation behaviour of the bushcricket *Pachysagella australis* Walker (Orthoptera, Tettigoniidae, Saginae).. Anim Behav.

[48] Rheinlaender J, Shen J-X, Römer H (2006). Auditory lateralization in bushcrickets: a new dichotic paradigm.. J Comp Physiol A.

[49] von Helversen D, Lehrer M (1997). Acoustic communication and orientation in grasshoppers.. Orientation and communication in Arthropods.

[50] Shen J-X, Feng AS, Xu Z-M, Yu Z-L, Arch VS, Yu X-J, Narins PM (2008). Ultrasonic frogs show hyperacute phonotaxis to female courtship calls.. Nature.

[51] Knudsen EI, Blasdel GG, Konishi M (1979). Sound Localization by the barn owl (*Tyto alba*) measured with the search coil technique.. J Comp Physiol A.

[52] Heffner RS, Koay G, Heffner HE (2007). Sound-localization acuity and its relation to vision in large and small fruit-eating bats: I. Echolocating species, *Phyllostomus hastatus* and *Carollia perspicillata*.. Hearing Research.

[53] Mills AW (1958). On the minimum audible angle.. J Acoust Soc Am.

[54] Simmons JA, Kick SA, Lawrence BD, Hale C, Bard C, Escudi B (1983). Acuity of horizontal angle discrimination by the echolocating bat, *Eptesicus fuscus*.. J Comp Physiol A.

[55] Heffner RS, Heffner HE (1982). Hearing in the elephant (*Elephas maximus*): Absolute sensitivity, frequency discrimination, and sound localization.. J Comp Physiol Psychol.

[56] Renaud DL, Popper AN (1975). Sound localization by the bottle nose porpoise *Tursiops truncatus*.. J Exp Biol.

